# Protective Potential of Vinpocetine on Acrylamide Induced Liver Toxicity in Male Albino Rats

**DOI:** 10.1002/jbt.71039

**Published:** 2026-07-27

**Authors:** Eman M.S. Shaheen, Marwa A. E. Abd El‐Maksoud

**Affiliations:** ^1^ Zoology Department Faculty of Science, Benha University Benha Egypt

**Keywords:** acrylamide, oxidative stress and hepatic toxicity, tumor necrosis factor, vinpocetine

## Abstract

Acrylamide is a highly reactive carbonyl compound extensively utilized in industrial applications. It shows a significant association with oxidative stress, neurotoxic, and genotoxic effects. This study sought to assess the protective effects of vinpocetine, a synthetic analog of the natural alkaloid vincamine, which exhibits strong antioxidant and anti‐inflammatory properties, against ACR‐induced hepatic injury. Experimental animals were categorized into four groups and orally treated as follows: control group, Vinpo. group (5 mg/kg bw), Acrylamide group (38.27 mg/kg bw), and Vinpo. plus acrylamide group. Following the experimental period, biochemical analyses revealed that ACR administration significantly elevated hepatic marker enzymes and lipid profile as well as increased oxidative stress markers such as malondialdehyde (MDA) and tumor necrosis factor (TNF‐α) immunoexpression levels, with concomitant reductions in antioxidant defenses (catalase “CAT” and superoxide dismutase “SOD”). Conversely, co‐administration of vinpocetine mitigated the ACR‐induced alterations and helped in the normalization of biochemical parameters, enhancing antioxidant capacity and attenuation of histopathological damage and apoptotic processes.

## Graphical abstract

This study sought to assess the protective effects of vinpocetine, a synthetic analog of the natural alkaloid vincamine exhibiting strong antioxidant and anti‐inflammatory properties against ACR‐induced hepatic injury. Co‐administration of vinpocetine mitigated the ACR‐induced alterations and help in normalization of biochemical parameters, enhancing antioxidant capacity and attenuation of histopathological damage and apoptotic processes.

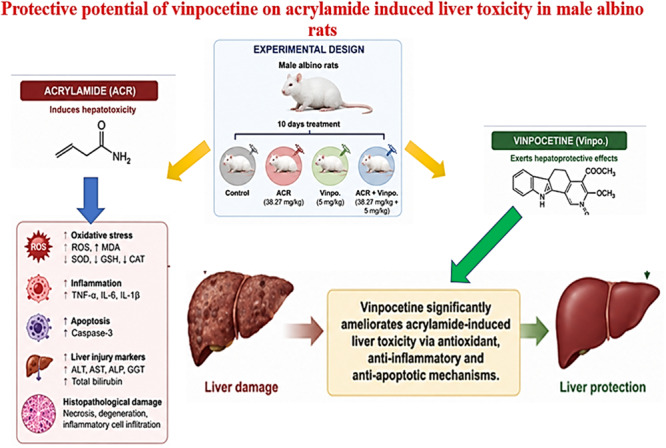

## 1 Introduction

Vinpocetine (vinpo.) is an alkaloid used worldwide as dietary supplements. It is derived from the leaf tissues of the *Vinca minor* periwinkle plant [1]. Vinpocetine exerts antiapoptotic effects and antioxidant effects by attenuating the generation of reactive oxygen species (ROS), decreasing lipid peroxidation and neutralizing free radicals [2]. Also, it has an analgesic effect by attenuating pain responses and an antidepressant effect in various experimental animals [3]. Vinpocetine acts as a vasodilator that augments cerebral perfusion and promotes glucose and oxygen uptake, resulting in improved metabolism and stimulation of adenosine triphosphate (ATP) synthesis [4].

Acrylamide (ACR) is an organic amide, an odorless crystalline solid at room temperature, naturally present in roasted coffee, potatoes, cereals, cocoa, and in cigarette smoke [5]. Thermal processing of foods leads to acrylamide formation through the Maillard reaction, where asparagine reacts with reducing sugars [6]. Within the body metabolism, ACR converts to glycidamide, a reactive metabolite that accumulates in many organs such as kidney and liver, causing nephrotoxic and hepatotoxic effects. ACR has the potential to compromise cellular antioxidant capacity through its ability to elevate ROS and apoptosis rates and release pro‐inflammatory mediators [7]. ACR exerted hepatotoxic and nephrotoxic effects through the induction of DNA injury, autophagic dysregulation, mitochondrial impairment, and histomorphology alterations in renal nephrons and hepatic cells [8].

Previous studies have investigated the toxic effects of acrylamide and the pharmacological activities of vinpocetine separately. However, limited data are available regarding the potential protective effects of vinpocetine against acrylamide‐induced organ toxicity, particularly hepatic injury. Therefore, the present study was designed to investigate, for the first time, the hepatoprotective, antioxidant, and anti‐inflammatory effects of vinpocetine in acrylamide‐treated animals.

## 2 Materials and Methods

### 2.1 Chemical Compounds and Pharmaceutical Agents

Vinpocetine was supplied as a dietary supplement by PHARCO Pharmaceuticals Company (Egypt). Acrylamide powder was purchased from Alpha Chemika (India).

### 2.2 Experimental Animals

The animals (28 male albino rats) were obtained from the Laboratory Animal Unit, Helwan Farm–VACSERA, Egypt and weighted 130 ± 10 g. Rats were randomly distributed into experimental groups and housed under standardized environmental conditions (adequate ventilation, a constant temperature of 22°C, a 12 h light/12 h dark photoperiod, and unrestricted access to food and water.) for 1 week before the initiation of the experiment.

The Institutional Animal Care and Use Committee (IACUC) of the Zoology Department, Science Faculty, Benha University was reviewed, approved the experimental protocol of the current study under No.: ZD/FSc/BU‐IACUC/2022‐16e.

### 2.3 Animal Groups

Animals were randomly assigned in equal numbers to four experimental groups as described below:

Control group: Rats administered by 0.5 mL of saline.

Vinpo. group: animals administered orally at a dose of Vinpo (5 mg/kg bw) according to [9]

ACR group: Rats were administered oral dose of AC (38.27 mg/kg bw) according to [10].

Vinpo. plus ACR group: Rats were administered Vinpo. and, after 30 min, received a dose of acrylamide.

All treatments were delivered orally by gavage. Ten days later (the experimental period), blood and liver samples were collected from each rat for biochemical evaluation, histological, and immunohistochemical analyses.

Preparation of biological samples and biochemical assessment:

Blood samples were withdrawn from anesthetized rats and then centrifuged to separate sera. These sera were subsequently used to determine levels of lipid profile parameters (triglycerides [TG], total cholesterol [TC], low‐density lipoprotein cholesterol [LDL‐C], and high‐density lipoprotein cholesterol [HDL‐C]), levels of hepatic enzymes, including alanine aminotransferase (ALT), gamma‐glutamyl transferase (GGT), alkaline phosphatase (ALP), and aspartate aminotransferase (AST), total bilirubin (T. Bili.), direct bilirubin (DB) and glucose level using spectrophotometric assays with BioSystems (Spain) commercial kits. Very low‐density lipoprotein cholesterol [VLDL‐C] and atherogenic index were calculated.

Liver samples were excised from each rat and rinsed with phosphate‐buffered saline (PBS). Liver homogenates were prepared by homogenizing 10 mg of tissue in PBS, then centrifuged to obtain homogenates that were used to assess hepatic oxidative stress markers, such as catalase (CAT), superoxide dismutase (SOD), and malondialdehyde (MDA) by using spectrophotometric assays with BioVision (USA) commercial kits.”

Examination of histopathological and immunohistochemical:

By following the method of [11], paraffin‐embedded liver tissue sections were first deparaffinized in xylene and rehydrated through a graded series of ethanol solutions and sectioned at a thickness of 5 μm. The sections were subsequently deparaffinized and rehydrated, and a subset was stained with hematoxylin and eosin (H&E) for histological examination.

Other liver sections were incubated with antigen retrieval solution, followed by treatment with hydrogen peroxide to inhibit activity of endogenous peroxidase and blocking with bovine serum albumin to prevent non‐specific binding.

After blocking, the sections were incubated overnight at 4°C with a primary antibody against tumor necrosis factor‐alpha (TNF‐α), diluted according to the manufacturer's recommendations. Following incubation, sections were thoroughly washed with phosphate‐buffered saline (PBS) to remove unbound primary antibody. Thereafter, sections were incubated with an appropriate horseradish peroxidase (HRP)‐conjugated secondary antibody at room temperature for 30–60 min. Finally, sections were counterstained, dehydrated through graded alcohols, cleared in xylene, and mounted using a suitable mounting medium for subsequent microscopic examination. The percentage area of immunoreactivity in hepatic tissue was quantitatively analyzed using Image‐Pro Plus software (Media Cybernetics, USA).

Statistical analysis:

Data were processed and analyzed using SPSS statistical software. (Version 24.0). Group means and standard deviations (M ± SD) were calculated based on seven replicates per group. Group comparisons were performed using one‐way ANOVA, followed by Duncan's multiple‐range test at a significance difference of *p* < 0.05. Graphical representations were generated using Sigma Plot software (version 14.0).”

## 3 Results

### 3.1 Hepatic Enzyme and Function Indicators

Administration of ACR resulted in a marked (*P* ˂ 0.05) increase in the liver function biomarkers (AST, ALT, ALP, GGT, DB, and T. Bili.) as compared to all other groups. The hepatic enzyme and function indicators of the Vinpo. plus ACR group was significantly decrease compared to ACR group (Table 1).

**Table 1:** Effect of acrylamide (ACR), Vinpocetine (Vinpo.) and Vinpo. plus ACR on liver function biomarkers in male albino rats.

	Control	Vinpo.	ACR	Vinpo. plus ACR
AST (U/L)	139.45 ± 0.77^c^	141.00 ± 1.41^c^	163.65 ± 1.90^a^	155.85 ± 0.21^b^
ALT (U/L)	35.90 ± 1.27^c^	37.50 ± 0.71^c^	59.35 ± 0.92^a^	40.50 ± 0.71^b^
ALP (U/L)	173.00 ± 1.41^c^	153.90 ± 0.14^d^	251.80 ± 1.55^a^	218.95 ± 1.48^b^
GGT (U/L)	11.10 ± 0.28^d^	12.30 ± 0.42^c^	20.10 ± 0.28^a^	17.90 ± 0.42^b^
T. Bili. (mg/dl)	0.39 ± 0.05^c^	0.44 ± 0.06 b^c^	0.90 ± 0.02^a^	0.57 ± 0.04^b^
DB (mg/dl)	0.12 ± 01^b^	0.13 ± 0.01^b^	0.16 ± 0.01^a^	0.14 ± 0.01^b^

*Note:* Different letters mean there are significant different in the same row at *p* < 0.05.

Data represented as mean ± SD “standard deviation” for *n* = 7.

### 3.2 Lipid Levels

Animals treated with ACR showed significant (*P* ˂ 0.05) elevation in circulating serum levels of TG, TC, and LDL‐C and marked decrease in HDL‐C level compared to the other groups. The obtained data (table 2) of the Vinpo plus ACR group demonstrated a significant improvement in the serum lipid levels compared to the ACR group.

**Table 2:** Effect of acrylamide (ACR), Vinpocetine (Vinpo.) and Vinpo. plus ACR on lipid profile in male albino rats.

	Control	Vinpo.	ACR	Vinpo. plus ACR
TG (mg/dl)	109.45 ± 0.77^d^	118.50 ± 2.12^c^	158.15 ± 1.20^a^	146.60 ± 0.56^b^
TC (mg/dl)	79.50 ± 0.70^c^	81.45 ± 0.64^c^	164.35 ± 1.91^a^	101.00 ± 1.41^b^
HDL (mg/dl)	36.90 ± 0.14^a^	37.60 ± 0.85^a^	28.20 ± 0.56^c^	32.70 ± 0.42^b^
LDL (mg/dl)	20.00 ± 0.71^c^	21.10 ± 0.28^c^	30.75 ± 1.20^a^	27.35 ± 0.63^b^
VLDL (mg/dl)	21.89 ± 0.15^d^	23.70 ± 0.42^c^	31.63 ± 0.24^a^	29.32 ± 0.11^b^
Atherogenic Index	2.15 ± 0.23^c^	2.12 ± 0.028^c^	5.82 ±.05^a^	3.08 ± 0.08^b^

*Note:* Different letters mean there are significant different in the same row at *p* < 0.05.

Data represented as mean ± SD “standard deviation” for *n* = 7.

### 3.3 Oxidative Stress Markers

The group rats that were treated with ACR showed a significant (*p* ˂ 0.05) rise in MDA level and a significant reduction in activities of SOD and CAT compared with the other group. Administration of Vinpo. 30 min. early to ACR administration caused significant improved in hepatic oxidative stress markers (Table 3).

**Table 3:** Effect of acrylamide (ACR), Vinpocetine (Vinpo.) and Vinpo. plus ACR on oxidative stress markers in liver tissue homogenate in male albino rats.

	Control	Vinpo.	ACR	Vinpo. plus ACR
MDA nmol/10 mg tissue	1.93 ± 0.11^c^	1.83 ± 0.06^c^	6.31 ± 0.14^a^	4.43 ± 0.16^b^
SOD U/10 mg tissue	6.06 ± 0.03^a^	6.05 ± 0.22^a^	2.55 ± 0.32^c^	3.45 ± 0.08^b^
CAT mU/10 mg tissue	5.63 ± 0.13^a^	5.72 ± 0.33^a^	2.23 ± 0.13^c^	3.05 ± 0.09^b^

*Note:* Different letters mean there are significant different in the same row at *p* < 0.05.

Data represented as mean ± SD “standard deviation” for *n* = 7.

### 3.4 Blood Glucose Level

A significant (*p* < 0.05) increase and decrease in blood glucose levels was observed in ACR treated group and Vinpo plus ACR group respectively (Figure 1).

**Figure 1:**
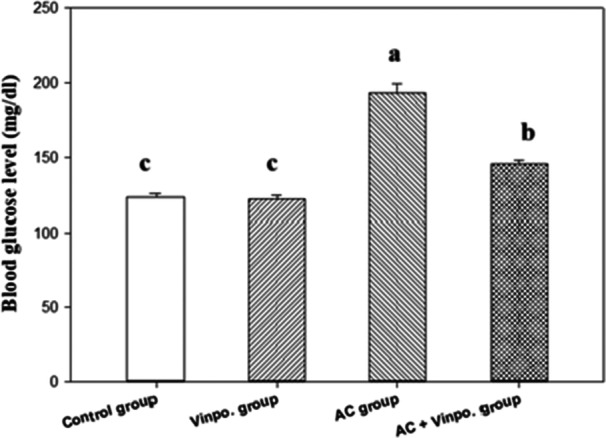
Effect of acrylamide (ACR), Vinpocetine (Vinpo.) and Vinpo. plus ACR on blood glucose level in male albino rats. Different letters mean there are significant different at *p* < 0.05. Data represented as mean ± SD “standard deviation” for *n* = 7.

### 3.5 Structural Histopathological Modifications of Liver Tissues

Microscopic examination of hepatic tissue sections in control and vinpocine. groups revealed normal hepatic architecture, with hepatocytes arranged in cords and possessing centrally located spherical nuclei with prominent nucleoli (Figure 2A). Occasional binucleated hepatocytes were detected in the livers of rats that received vinpocetine (Figure 2B). Conversely, histopathological examination of the livers from acrylamide‐intoxicated rats revealed severe dilatation and congestion of the portal and central veins with extensive hepatocellular degeneration, particularly in the centrilobular regions (Figure 2C). Multiple foci of hepatic necrosis, characterized by cytoplasmic eosinophilia and pyknosis of nuclei together with focal areas of hemorrhages, were widely distributed throughout the hepatic parenchyma and edema and bile ductal hyperplasia (Figures 2D, E & F). Treatment with vinpocetine 30 min. prior to ACR exposure alleviates the hepatic damage induced by acrylamide. The liver showed only dilatation of the central veins with focal areas of mild hepatic degenerative changes represented by cytoplasmic vacuolation of some hepatic cells (Figure 2G). Few inflammatory cells were observed aggregated in the portal areas. (Figure 2H).

**Figure 2:**
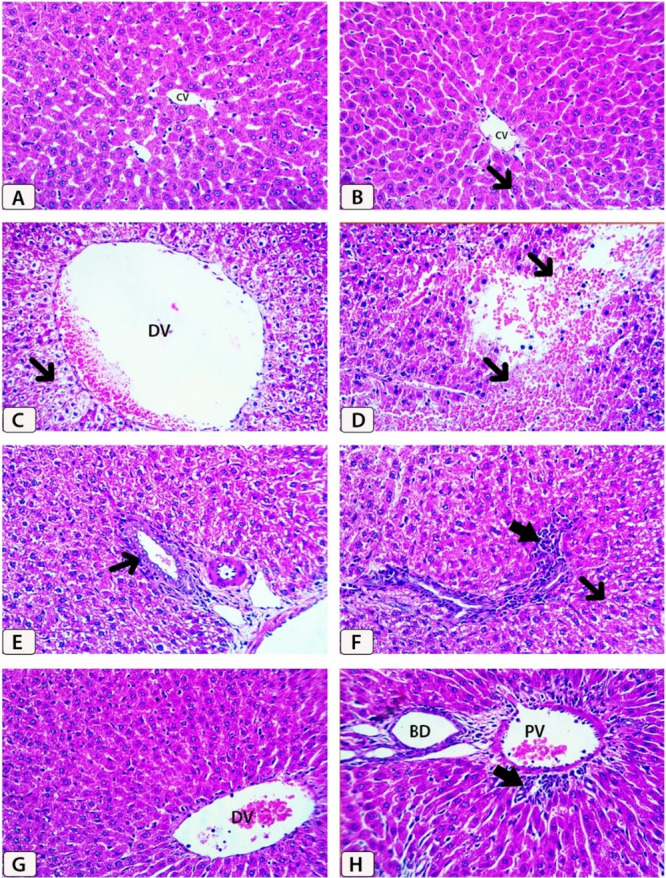
Photomicrograph (H & E stain X200) of the liver sections of rats in control group (A) showing rows of hepatocytes with centrally located spherical nucleus radiate from the central vein (CV) toward the periphery of the hepatic lobule. Vinpo. group (B) showing hepatocytes with few binucleated hepatic cells (arrow), AC group (C–F) showing severe dilatation of the central vein (DV) with centrolobular hepatic degeneration (arrow) (C), hepatic necrosis and hemorrhages (arrows) (D), bile ductal hyperplasia (arrow) (E) and mononuclear cellular infiltration of the portal area (thick arrow) and degeneration of some hepatic cells (thin arrow) (F). AC plus Vinpo. Group (G, H) showing dilatation of the central vein (DV) with mild vacuolation of some hepatic cells (G) and dilated bile duct (DB) and congestion of the portal vessel with aggregation of few inflammatory cells in the portal area (thick arrow) (H).

### 3.6 Immunohistochemical Evaluation

In the control group, immunohistochemical staining for TNF‐α in liver sections revealed minimal expression, localized primarily to the walls of the hepatic sinusoids (Figure 3A). In contrast, ACR administration caused a marked elevation in TNF‐α expression (Figure 3B). Figure 3D indicated that vinpocetine treatment prior to ACR resulted in marked decreases in the area percentage of TNF‐α expression.

**Figure 3:**
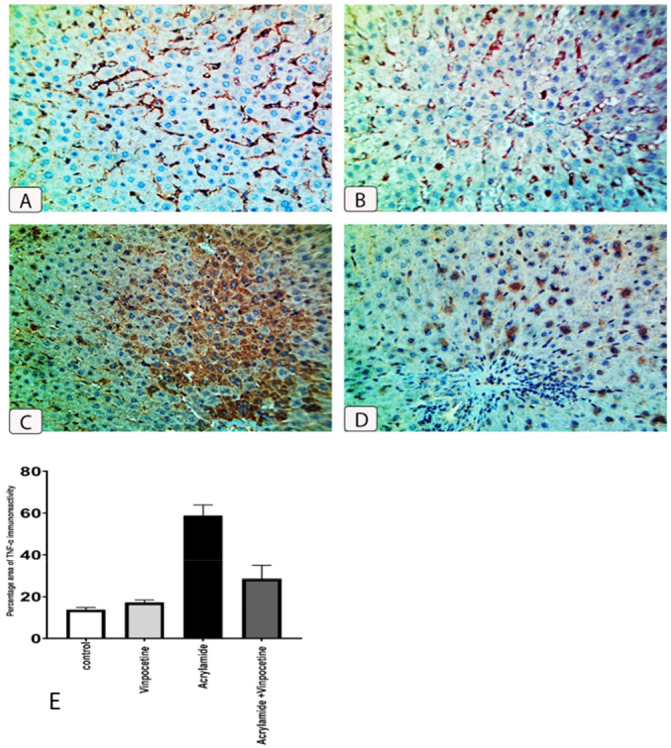
Immunohistochemical photomicrographs (A–D) TNF‐α expression in the hepatic tissues of control (A) Vinpo. (B) ACR (C) Vinpo. plus ACR (D) X200 Percentage area of TNF‐α immunoreactivity represent mean ± standard deviation (E).

## 4 Discussion

The liver is highly susceptible to toxic and harmful substances in experimental animals and humans. Exposure to ACR caused harmful alterations in liver tissues. As evidenced by previous experimental findings, ACR‐mediated hepatotoxicity which caused by an elevation in oxidative stress, inflammatory reactions, and impairment of antioxidant defense systems [12]. The current study indicated that ACR administration caused biochemical alterations in measured parameters (significant elevation in liver function parameters, serum circulating levels of TG, TC, LDL‐C, blood glucose, MDA, and a significant decline in HDL‐C level and antioxidant activities).

These biochemical alterations are associated with histopathological changes such as hepatocellular degeneration, infiltration of inflammatory cells, central vein congestion, and necrosis in the liver of exposed animals, supporting the occurrence of hepatotoxicity after ACR administration. Liver injury after ACR treatment is possibly attributable to promotion of oxidant–antioxidant imbalance, inflammation, and disturbance of redox balance. Also, it may be associated with modulation of some pathways such as cytochrome P450 2E1 and dependent metabolism or activation of proinflammatory signaling, which consequently augments hepatic damage and apoptotic processes [13].

Biochemical indicators of liver function, such as AST, ALT, and bilirubin, were markedly elevated after ACR treatment [12]. Data in the current study revealed that ACR caused an elevation in all measured hepatic function biomarkers (AST, ALT, ALP, GGT, T. Bili, and DB). These results agreed with [10, 14], who reported that ACR caused hepatocellular injury and increased membrane permeability, which helped enzymes to leak into circulation. Moreover, elevated bilirubin levels have been observed, reflecting impaired hepatic clearance and disruption of bile flow. These results were indicated by structural histopathological examination of the rat's hepatocyte exposed to ACR, which showed degenerative and vascular congestion associated with mild infiltration of mononuclear inflammatory cells.

Also, authors [15] reported that ACR exposure at different doses caused moderate mononuclear infiltration of inflammatory cells, vascular congestion, and widespread hepatocellular necrosis.

ACR generates ROS which promotes lipid peroxidation, as cellular fatty acids are oxidized by ROS and produce lipid peroxyl radicals. ACR exposure resulted in redox imbalance, characterized by increased lipid peroxidation, nitric oxide production, and formation of protein carbonyl [16]. In current research, alternation in antioxidant defense enzymes like CAT, SOD, and MDA were observed after ACR treatment. These results may be attributed to ACR exposure resulting in an elevation in ROS formation level by suppressing the activities of mitochondrial complexes [7]. In this study oxidative stress, inflammation, and histological changes in hepatocytes after ACR induction were confirmed by reduction in CAT and SOD activities and an elevation in MDA and TNF‐α immunoexpression levels. Additionally, acrylamide exposure significantly upregulated inflammatory cytokines, including IL‐1, TNF‐α, and IL‐6, via activation of the NF‐κB signaling pathway [17]. These results are consistent with previous reports by authors [18], who concluded that rats treated with acrylamide showed a significant increase in MDA level and a significant decrease in antioxidant capacities (GSH and SOD). These changes are considered an indicator of oxidative stress, which induces hepatocyte necrosis.

Authors [19] reported that ACR induces oxidative stress and inflammation, indicated by an alternation in hematological parameters: “decrease in erythrocyte count, hemoglobin amount, and hematocrit value.”

Data in the current study revealed that rats exposed to ACR showed a marked increase in blood glucose level. These results could be ascribed to the fact that ACR caused insulin resistance and increased oxidative stress on pancreas beta cells causing alternations in glucose metabolism and insulin signaling pathways [20]. These findings are consistent with authors [21, 22], who revealed that elevated levels of the hemoglobin adduct of ACR (HbAA) in humans were correlated with insulin resistance and reduction in insulin level. Also, authors [23] concluded that urinary metabolites after ACR treatment correlated with an elevation in fasting blood glucose (FBG) level in a dose‐responsive manner.

In this study, vinpocetine administration diminishes the biochemical alternation caused by ACR exposure in treated animals. This may be attributed to vinpo. improving insulin sensitivity and lipid metabolism, significantly lowering the elevation in ALT, AST, and ALP activities, ameliorating oxidative stress, and mitigating the histological hepatic injury caused by ACR. These results agreed with the authors [24]. who reported that vinpo. has anti‐atherogenic action. Vinpocetine acts for the inhibition of nuclear factor kappa B (NF‐κB) along with inflammatory mediators such as TNF‐α and IL‐6, ROS, and another inflammatory agent formation level. Also, vinpo. exerts anti‐inflammatory and free radical–scavenging activities against renal and hepatic damage and cerebral ischemia/reperfusion injury in diabetic animals [25, 26].

Vinpocetine exhibits potent antioxidant activities by scavenging hydroxyl radicals, inhibiting the generation of ROS and suppressing lipid peroxidation. Additionally, it mitigates free radicals' formation, thereby contributing to the attenuation of high glucose–induced oxidative stress and cellular damage [27].

Vinpocetine possesses multiple pharmacological actions, including vasodilator effects; inhibition of cyclic nucleotide phosphodiesterase 1 (PDE1), voltage‐gated sodium channels, and calcium channels; and increased glucose and oxygen utilization in the brain. In the current study, vinpocetine suppressed anti‐apoptotic markers such as TNF‐α. This may be attributed to the fact that Vinpocetine suppresses the release of TNF‐α–induced pro‐inflammatory mediators through blocking the IκB kinase (IKK)/NF‐κB signaling pathway [27].

The study results are parallel with the results of [28], who concluded that,there is a reduction in TNF‐α and caspase 3 activities after treatment with vinpocetine against cisplatin‐induced hepatic toxicity.

## 5 Conclusion

In summary, the current study revealed that vinpo. can improve the damage induced by ACR. We suggest that treatment with vinpo. possess hepatoprotective, redox‐modulating, anti‐inflammatory, and multi‐functional effects against deterioration effects triggered by ACR.

## Author Contributions

**Eman M.S. Shaheen:** formal analysis, data curation, methodology, writing – original draft, visualization, conceptualization. **Marwa A. E. Abd El‐Maksoud:** methodology, investigation, supervision, writing – review and editing, writing – original draft, visualization, data curation, software, conceptualization, formal analysis.

## Funding

The authors have nothing to report.

## Conflicts of Interest

The authors declare no conflicts of interest.

## Data Availability

The data that support the findings of this study are available on request from the corresponding author. The data are not publicly available due to privacy or ethical restrictions. The data supporting the findings in current study are available from the corresponding author upon reasonable request.
